# Typifications in neotropical Sapotaceae

**DOI:** 10.3897/phytokeys.170.54718

**Published:** 2020-12-21

**Authors:** Anderson Alves-Araújo, Quélita dos Santos Moraes, Renara Nichio-Amaral, Victor Santos Miranda

**Affiliations:** 1 Departamento de Ciências Agrárias e Biológicas, Universidade Federal do Espírito Santo-UFES, Rodovia BR 101 Norte, Km 60, São Mateus-ES, Brazil Universidade Estadual de Feira de Santana Feira de Santana Brazil; 2 Programa de Pós-Graduação em Botânica, Universidade Estadual de Feira de Santana, Av. Transnordestina s.n., Feira de Santana-BA, Brazil Universidade Federal do Espírito Santo-UFES São Mateus Brazil

**Keywords:** Ericales, historical botany, lectotypification, nomenclature

## Abstract

Sapotaceae is historically known as having a tricky and challenging taxonomy due to tangled morphologic heterogeneity. Consequently, this resulted in a large number of described genera and binomials. After Pennington’s Flora Neotropica work, several of those nomenclature issues were resolved. Nevertheless, many binomials remain unsolved and up for typification. Thus, following the International Code of Nomenclature for Algae, Fungi and Plants, we propose 74 new lectotype designations, four of these are second-step typifications.

## Introduction

Sapotaceae has 65–70 genera with around 1,250 species and is an important plant component from tropical regions in the world (Swenson et al. 2020). It is an economically interesting family by providing latex-derived products such as gutta-percha and chewing gum, valuable and durable timber and edible fruits (Pennington 1990, 1991).

In Linnaeus’ first edition of Species Plantarum (Linnaeus 1753), the author began the taxonomic history of Sapotaceae by describing six species: *Achras zapota* L. (= *Manilkara zapota* (L.) P. Royen), *Chrysophyllum cainito* L., *Mimusops elengi* L., *Mimusops kauki* L. (= *Manilkara kauki* (L.) Dubard), *Sideroxylon inerme* L. and *Sideroxylon spinosum* L. However, Jussieu’s Genera Plantarum (Jussieu 1789) was the first work that recognised it as a homogenous group, then called “Sapotae”. Since then, many morphological classification proposals have been reported, such as Baehni (1938, 1965), Lam (1939), Aubréville (1964) and Pennington (1990, 1991). Currently, based on phylogenetic and morphological analysis, Sapotaceae is divided into three monophyletic subfamilies: Sarcospermatoideae, Sapotoideae and Chrysophylloideae (Swenson and Anderberg 2005).

Historically, Sapotaceae taxonomy can be considered as tricky and truly challenging due to the morphologic heterogeneity of its many genera and species. This resulted in a large number of described genera and binomials associated with morphologically-related species that have tangled circumscriptions, such as *Pouteria* Aubl. and *Chrysophyllum* L. However, these numbers may often vary according to recent new discoveries (Alves-Araújo and Alves 2011, 2012a, b; Popovkin et al. 2016; Alves-Araújo and Mônico 2017; Sossai et al. 2017; Alves-Araújo 2018a), re-circumscriptions (Swenson et al. 2007; Mackinder et al. 2016) and phylogenetic studies (Terra-Araújo et al. 2015; Faria et al. 2017). *Pouteria* and *Chrysophyllum* are the largest genera in Sapotaceae with around 270 and 100 accepted names, respectively (The Plant List 2013). Furthermore, for tropical Americas, they have together more than 60 synonyms, most of them applied to *Pouteria* (around 45) (Pennington 1990, 1991).

Efforts, focusing on Sapotaceae internal relationships, were raised in the past two decades aiming to clarify genera and species boundaries (Swenson and Anderberg 2005; Swenson et al. 2008, 2013; Terra-Araújo et al. 2015; Faria et al. 2017). In addition, better understanding of taxonomic delimitation for many species, or even infra-species categories, are the goals for some available works (Terra-Araújo et al. 2012, 2016; Alves-Araújo et al. 2014; Alves-Araújo 2018b; Ferreira et al. 2019). Fortunately, those recent works had, as background, one of the most important contributions for the family in the world: Pennington’s Flora Neotropica (Pennington 1990).

In his work, Pennington (1990) brings several aspects, from palynology (Harley 1990) to general taxonomy, approaching 11 genera and almost 1/3 of the world richness for Sapotaceae (approximately 400 species). Taxonomically, the author provides substantial historical information about those binomials and their *typi*, complementary analysed vouchers, geographic distribution maps and illustrations, keys and descriptions of many new species. He also included new synonyms and performed some typifications.

While performing Sapotaceae studies in Brazil and after consulting Flora Neotropica, despite Pennington’s extraordinary efforts, we realised many binomials remain up for typification processes according to the International Code of Nomenclature (ICN) criteria (Turland et al. 2018). Thus, we aim to contribute for nomenclature stability through typification of the untypified names by choosing/indicating lectotypes when needed.

## Material and methods

This synopsis is based on the examination of binomials cited by Pennington (1990) in Flora Neotropica: Sapotaceae. To do so, information about protologues, herbarium specimens and historic and relevant literature with details of original elements were gathered together to perform the typifications. Main databases, such as IPNI (International Plant Names Index, http://www.ipni.org), Reflora (http://reflora.jbrj.gov.br/), SpeciesLink (http://www.splink.org.br/index?lang=pt), The Plant List (http://www.theplantlist.org) and Tropicos (https://www.tropicos.org/home) for the names herein treated and JSTOR Global Plants (https://plants.jstor.org/) for digitised plant specimens, have been consulted in order to analyse vouchers housed in the herbaria A, B, BM, BR, C, CORD, E, F, G, GH, GOET, HAL, HBG, IAN, K, L, LD, LE, LL, M, MA, MEL, MG, MICH, MIN, MO, MPU, NY, OXF, P, RB, S, SI, TCD, U, UC, UPS, US, W and YU (acronyms following Thiers, continuously updated).

All the newly-proposed types were carefully checked and are in accordance with the Articles 9.11, 9.12, 9.13 and 9.22 of the ICN adopted in Shenzhen (Turland et al. 2018). We also provide the homotypic synonyms and newly-tracked samples for accepted names with all available herbaria barcodes or collection numbers.

Pertinent details of some type designation were suppressed due to the amount of type designations. Nevertheless, we established that, to be eligible, the vouchers should be well-preserved and exhibit reproductive and/or diagnostic characteristics. There are three different situations (hereafter coded by numbers) that led us to typify: (1) Holotype not designated in the protologue; (2) If designated, there is more than one sample in the same herbarium or syntypes; and (3) Holotype destroyed or missing.

The lectotype designations take into account, depending on the context, the author’s original institution, the collector’s original institution and information from the labels. All lectotypes are formatted as follows: currently accepted name in each entry is shown alphabetically in bold italic typeface with full bibliographic reference, basionym (when present), any homotypic name, designation of lectotype and code for situation in bold typeface (1, 2 or 3) and any exceptional supporting notes. For those cases in which lectotypes were chosen for synonyms, they are presented below the currently accepted binomial with the same previously cited format, except by being preceded by “=” and only having italic typeface.

Type collections, including those cited by Pennington (1990), where we have not tracked or seen material are indicated by “n.v.”. Additional information from herbarium labels are presented between square brackets “[...]”.

## Typifications and new combinations

We here provide a list of the species names in seven genera, 74 new lectotype designations [10 for code (1), 42 for code (2) and 22 for code (3)], four of these being second-step typifications.

**Chrysophyllum argenteum Jacq. subsp. argenteum , Enum. syst. pl. 15. 1760. Type: (lectotype, designated by Pennington [1990: 543]: Jacquin, Select. stirp. amer. hist. t. 38, f. 1. 1763).**

= *Sideroxylon guadalupense* Spreng., Syst. veg. 1: 666. 1824. Type: Guadeloupe, *C. Bertero s.n.* (lectotype, designated by Pennington [1990: 544]: MO [n.v.]; isolectotypes: P [barcodes P00644517, P00644518, P00649285]!).

= *Chrysophyllum immersum* Urb., Repert. Spec. Nov. Regni Veg. 15: 414. 1919. Type: Tobago. Easterfield, (yfr), *W. Broadway 4411* (lectotype, designated here: P [barcode P00649287]!; isolectotypes: BM [barcode BM000952601]!, F [barcode V0071916F]!, G [barcode G00434800]!, L [barcodes L 0064667, L 0064668]!, NY [barcode 00273428]!, P [barcode P00649288]!, U [barcode U 1596320]!, US [barcode 00323626]!). **(3)**

**Chrysophyllum argenteum subsp. auratum (Miq.) T.D.Penn., Fl. Neotrop. Monogr. 52: 545–547, f. 126F–J. 1990.**

≡ *Chrysophyllum auratum* Miq. in Martius Fl. Bras. 7: 97–98. 1863. Type: Guyana. Nr. Roraima, 1842–1843, (fl), *Rob. Schomburgk [ser. 2] 864* (lectotype, designated here: K [barcode K000633985]!; isolectotypes: BM [barcode BM000952599]!, F [barcodes V0071922F, V0071923F – fragm., V0071924F, V0071925F – fragm.]!, GH [barcode GH00075567]!, P [barcodes P00649294, P00649295, P00649296]!, US [barcode 00323621]!). **(2)**

= Chrysophyllum auratum var. majus Miq. in Martius, Fl. Bras. 7: 98. 1863. Type: Guyana. Nr. Roraima, [1842–1843], (fl), *Rob. Schomburgk [ser. 2] 813* (lectotype, designated here: BM [barcode BM0952598]!; isolectotypes: G [barcode G00434798 – mounted on three herbarium sheets]!, P [barcodes P00649291, P00649292, P00649293]!, U [barcode U 0006569]!). **(2)**

**Note 1.**Miquel (1863) mentioned the type collection of *Chrysophyllum auratum* Miq. as being composed of two different collections: Schomburgk #864 and #1389. Some of the original materials bring both collector numbers on their labels. According to van Dam (2002), Robert Schomburgk’s collection may have two numbers on the labels where the first one corresponds to his own collection and the second is related to his brother’s collection, Richard Schomburgk. Based on those samples, *Chrysophyllum auratum* was re-circumscribed as subspecies of *Chrysophyllum argenteum* Jacquin by Pennington (1990) under epithet C. argenteum subsp. auratum (Miq.) T.D.Penn.

**Note 2.**Miquel (1863) mentioned the type collection of Chrysophyllum auratum var. majus under Schomburgk #813 and #1507. As explained above, only #813 corresponds to Robert Schomburgk’s collection (van Dam 2002). Labels from those samples housed at BM, G, P (3 sheets) and U herbaria have solely the annotation #813, but those ones from K show #1507 (K000633986) or both #813 and #1507 (K000633987). We assumed only the vouchers under #813 and selected the sample at BM (BM0952598) as lectotype by its having well-preserved branches and flowers.

**Chrysophyllum argenteum subsp. ferrugineum (Ruiz & Pav.) T.D.Penn., Fl. Neotrop. Monogr. 52: 547–548. 1990.**

≡ *Nycterisition ferrugineum* Ruiz & Pav., Fl. peruv. 2: 47, pl. 187. 1799. Type: Peru. [Cuchero, Chichao & Puente de Pillao], [1787] (fl), *H. Ruiz & J. Pavón s.n.* (lectotype, designated here: MA [barcode MA814291]!; isolectotypes: BC [barcode BC873102 – mounted on three herbarium sheets]!, BR [barcode 0000005417438]!, F [barcodes V0042273F, V0042274F]!, G (barcode G00434794 – mounted on three herbarium sheets]!, HAL [barcode 0116317]!, M [barcode M0174554 – fragm.]!, MA [barcodes MA814287, MA814293, MA817957, MA817959]!, P [barcodes P00649297, P00649298 – mounted on three herbarium sheets, P00649299]!, TUB [n.v.]). **(2)**

***Chrysophyllum inornatum* Mart., Flora 21, Beibl. 2: 96. 1838 (reprinted as Herb. Fl. Bras. 176).**

*Chrysophyllum inornatum* Mart., Flora 21, Beibl. 2: 96. 1838 (reprinted as Herb. Fl. Bras. 176). Type: Brazil. [Rio de Janeiro, São Paulo or Minas Gerais], [1820] (fl.), *F. Sellow s.n. [4530*] (lectotype, designated here: G [barcode G00434783]!; isolectotypes: K [barcode K000653001]!, M [barcode M0174545]!, P [barcode P00649335]!, U [barcode U 0006575]!, UC [barcode UC158699, UC493209]!, US [barcode 00037051]!). B†, F neg. 4247 **(2)**.

**Note.** The label of the specimen at M [M0174545] brings the collection date as 1820. According to Moraes (2008), the set of specimens were most probably collected in Sellow’s third journey in the Rio de Janeiro, São Paulo or Minas Gerais States.

**Chrysophyllum marginatum (Hook. & Arn.) Radlk. subsp. marginatum, Act. Congr. Bot. Anvers 1885: 170. 1887.**

≡ *Myrsine marginata* Hook. & Arn., J. Bot. 1: 283. 1834. Type: Brazil. Rio Grande do Sul: Porto Alegre, (fl), *J. Tweedie s.n.* (lectotype, designated by Pennington [1990: 561]: K [barcode K000633995]!).

= *Chrysophyllum maytenoides* [*maytenodes*] var. tenue Kuntze, Revis. gen. pl. 3(2): 194. 1898. Type: “South Paraguay”, Sep 1892 (yfr), *C. Kuntze s.n.* (lectotype [first-step designated by Pennington [1990: 561], as “isotype”], second-step designated here: NY [barcode 00273433]!; isolectotype: NY [barcode 00273432]!). **(2)**

**Chrysophyllum oliviforme L. subsp. oliviforme , Syst. Nat. (ed. 10) 2: 937. 1759. Type: (lectotype, designated by Vink [1958: 28]): Plumier, ed. Burmann, Gen. 57, tab. 69. 1756).**

= Chrysophyllum oliviforme var. pallescens Urb. in Pierre & Urban, Symb. Antill. 5: 157. 1904. Type: Haiti. (fl.), *L. Picarda 542* (lectotype, designated here: GH [barcode GH00075585]!; isolectotype: NY [barcode 00099909 – fragm.]!). **(3)**

= Chrysophyllum oliviforme var. platyphyllum Urb. in Pierre & Urban, Symb. Antill. 5: 157. 1904. Type: Haiti. R. Artibonite, (fl.), *L. Picarda 1642* (lectotype, designated here: NY [barcode 00099910]!; isolectotype: GH [barcode GH00075586]!). **(3)**

= *Chrysophyllum brachycalyx* Urb., Symb. Antill. 7: 327. 1912. Type: Jamaica. St. Elizabeth, Nr. Lacovia and Black River, (fl.), *W. Harris 9955* (lectotype, designated here: BM [barcode BM000952606]!; isolectotypes: F [barcode V0071915F]!, NY [barcode 00099906]!). **(3)**

= *Chrysophyllum gonavense* Urb., Ark. Bot. 22A (17): 74. 1929. Type: Haiti. Gonave Is., betw. Les Abricots and Les Etroits, (yfl.), *E. Ekman H8769* (lectotype, designated here: S [barcode S05-2188]!; isolectotype: S [barcode S05-2189 – mounted on two herbarium sheets]!). **(3)**

= *Chrysophyllum miragoaneum* [*miragoanum*] Urb., Ark. Bot. 22A (17): 75. 1929. Type: Haiti. Massif de la Hotte, Gros Mome Rochelois, nr. Miragoane, (fl.), *E. Ekman H8648* (lectotype, designated here: S [barcode S05-2204]!; isolectotypes: F [barcode V0071917F]!, G [barcode G00434772]!, GH [barcode GH00075584]!, K [barcode K000633983]!, NY [barcode 00099907]!, S [barcode S05-2206]!, US [barcodes 00782536, 00113123]!). **(3)**

**Chrysophyllum oliviforme subsp. angustifolium (Lam.) T.D.Penn., Fl. Neotrop. Monogr. 52: 541–543. 1990.**

≡ *Chrysophyllum angustifolium* Lam., Tabl. encycl. 2: 44. 1794. Type: Haiti. (f1.), *J. Martin 163* (holotype: P-LA [barcode P00649370]!).

= *Chrysophyllum picardae* Urb. in Pierre & Urban, Symb. Antill. 5: 158. 1904. Type: Haiti. Nr. Port-au-Prince, (fr.), *L. Picarda 1198* (lectotype, designated by Pennington [1990: 541]: P [barcode P00649369]!).

= *Chrysophyllum brachystylum* Urb., Symb. Antill. 7: 327. 1912. Type: Dominican Republic. Barahona, (fl.), *M. Fuertes 629* (lectotype, designated here: BM [barcode BM000952603]!; isolectotypes: BR [barcode 005416813]!, K [barcode K000633982]!). **(3)**

= *Chrysophyllum montanum* Urb., Repert. Regni Veg. 13: 469. 1915. Type: Dominican Republic. Barahona, Nr. Rincón, (fl.), *M. Fuertes 1296* (lectotype, designated here: BM [barcode BM000952604]!; isolectotypes: A [barcode A00075552]!, F [barcode V0071918F]!, G [barcode G00434773 – mounted on two herbarium sheets]!, GH [barcode GH00075551]!, HBG [barcode HBG510658]!, MICH [barcode MICH1104635]!, NY [barcode 00099908]!, P [barcode P00649372]!, S [barcode S05-10744]!, U [barcode U0006577]!). **(3)**

= *Chrysophyllum heterochroum* Urb., Ark. Bot. 22A (17): 74. 1929. Type: Haiti. Massif de la Selle, Gros Morne des Commissaires, nr. Anses-à-Pitre, (fl.), *E. Ekman H6748* (lectotype, designated here: S [barcode S05-2190]!; isolectotypes: S [barcode S05-2191]!, US [barcodes 00067586, 00782544]!). **(3)**

***Chrysophyllum revolutum* Mart. & Eichler in Miq., Fl. Bras. 7: 104. 1863.**

*Chrysophyllum revolutum* Mart. & Eichler in Miq., Fl. Bras. 7: 104. 1863. Type: Peru. San Martin, Nr. Tarapoto, 1855–1856 (fl, fr), *R. Spruce 4260* (lectotype, designated here: K [barcode K000653005]!; isolectotypes: BM [barcodes BM000952585, BM000795552 – fragm.]!, BR [barcode 005417445]!, E [barcode E00259471]!, F [barcodes V0042261F, V0042262F – fragm.]!, G [barcodes G00439209, G00439210]!, GH [barcode GH00075583]!, GOET [barcode GOET010914]!, K [barcodes K000653003, K000653004]!, MPU [barcode MPU019050]!, NY [barcode 00273445]!, OXF [n.v.], P [barcode P00649388]!, W [barcode 1889-0105047]!). **(2)**

**Note.** All type collection is labelled with Spruce #4260; however, Martius & Eichler in Miquel (1863) indicated them under Spruce #2460. This, most likely, could be a misunderstanding, based on a typing or writing error. Moreover, after searching for more vouchers, we found a different sample at TDC herbarium under Spruce #2460 that corresponds to *Peperomia macrostachya* C.DC. (Piperaceae) (TCD0007412), reinforcing our assumption.

***Chrysophyllum sparsiflorum* Klotzsch ex Miq. in Martius, Fl. Bras. 7: 90. 1863.**

*Chrysophyllum sparsiflorum* Klotzsch ex Miq. in Martius, Fl. Bras. 7: 90. 1863. Type: Guyana. Nr. Pirara, Jul-Aug 1843 (fl), *Rob. Schomburgk [ser. 2] 420* (lectotype, designated here: K [barcode K000653012]!; isolectotypes: F – fragm. [n.v.], K [barcode K000653011]!, P [barcode P00649397]!). **(2)**

= Chrysophyllum sparsiflorum var. fagifolium Miq. in Martius, Fl. Bras. 7: 90. 1863. Type: Brazil. Para: Nr. Santarem, *R. Spruce 632* (lectotype, designated here: M [barcode M0174521]!; isolectotype: U [barcode U 0006582]!). **(2)**

**Note.**Miquel (1863) mistakenly cited the samples collected by Richard Schomburgk # 680 as type collection of *Chrysophyllum sparsiflorum*. However, this set was collected, in fact, by Robert Schomburgk in his second collection series under #420 (van Dam 2002).

***Ecclinusa ramiflora* Mart., Flora 22, Beibl. 1: 2. 1839. Type: Brazil. Bahia: Ilhéus, *C. Martius s.n.* (holotype: M [barcode M0174559]!; isotypes: P [barcode P00649415]!).**

= *Ecclinusa abbreviata* Ducke, Bull. Mus. Hist. Nat. (Paris), sér. 2, 4: 743. 1932. Type: Brazil. Amazonas: Manaus, (fl.), *A. Ducke s.n.* (RB n° 22249) (lectotype, designated here: S [barcode S05-2174]!; isolectotypes:, G [barcode G00439194]!, K [barcodes K000640467, K000640468]!, P [barcode P00649414]!, RB [barcode RB00772244]!, U [barcode U 0006591]!, US [barcodes 00037025, 00037026]!). **(2)**

***Ecclinusa ulei* (K.Krause) Gilly ex Cronquist, Bull. Torrey Bot. Club 73: 311. 1946.**

≡ *Chrysophyllum ulei* K.Krause Notizbl. Königl. Bot. Gart. Berlin 6: 171. 1914. Type: Guyana. Roraima region, (fl.), *E. Ule 8729* (lectotype, designated here: K [barcode K000640466]!; isolectotype: MG [n.v.]). B†, F neg. 4258. **(3)**

***Ecclinusa lanceolata* (Mart. & Eichler ex Miq.) Pierre, Not. bot.: 57. 1891.**

≡ *Passaveria lanceolata* Mart. & Eichler ex Miq. in Martius, Fl. Bras. 7: 86. 1863. Type: Brazil. Amazonas: Rio Vaupes, Panure, 1852 (fl.), *R. Spruce 2639* (lectotype, designated here: K [barcode K000640463]!; isolectotypes: BR [barcode BR005417827]!, F [barcode V0072122F]!, K [barcodes K000640464, K000640465]!, P [barcodes P00649410, P00649411]!, TCD [barcode TCD0017840]!, W [barcode W-Rchb. n° 1889-0010203]!). **(2)**

***Ecclinusa lancifolia* (Mart. & Eichler ex Miq.) Eyma, Recueil Trav. Bot. Neerl. 33: 202. 1936.**

≡ *Passaveria lancifolia* Mart. & Eichler ex Miq. in Martius, Fl. Bras. 7: 86. 1863. Type: Brazil. Amazonas: R. Negro, Barcelos & San Isabel, above Barraroa, (fl.), *R. Spruce 1949* (lectotype, designated here: K [barcode K000640459]!; isolectotypes: BM [barcodes BM000952580, BM000952581, BM000952582]!, BR [barcode BR005417490]!, E [barcode E00259470]!, F [barcodes V0072123F, V0072124F]!, G [barcodes G00439195, G00439196]!, GH [barcode GH00075605]!, GOET [barcode GOET010916]!, K [barcode K000640460]!, NY [barcode 00273607]!, P [barcodes P00649412, P00649413]!, W [barcode 1889-0092142]!). **(2)**

**Manilkara jaimiqui subsp. emarginata (L.) Cronquist, Bull. Torrey Bot. Club 73: 467. 1946.**

≡ *Sloanea emarginata* L., Sp. pl. 512. 1753. Type: Bahama Archipelago, (fl), Herb Sloane vol. 232: 15 (Catesby 11: 87) (lectotype, designated by Dandy [1958: 112]: BM [barcode BM000952455]!; isolectotype: BM [barcode BM000952456]!).

= *Mimusops floridana* Engl., Bot. Jahrb. Syst. 12: 524. 1890. Type: USA. Florida: Boca Chica Key, *A. Curtiss 1766* (lectotype, designated here: BM [barcode BM001024778]!; isolectotypes: F [n.v.], G [barcode G00439225]!, M [barcode M0174507]!, MEL [barcode MEL 2466140A]!, NY [n.v.], P [barcode P00645516]!, US [barcodes 02706398, 02706420]!). **(3)**

**Manilkara jaimiqui subsp. wrightiana (Pierre) Cronquist, Bull. Torrey Bot. Club 73: 467. 1946.**

≡ *Mimusops wrightiana* Pierre in Pierre & Urban, Symb. Antill. 5: 171. 1904. Type: Cuba. (fl), *C. Wright 2917* (lectotype, designated by Pennington [1990: 92]: P [barcode P00645520]!; isolectotypes: G [barcode G00439221]!, GOET [barcode GOET010922]!, MO [barcodes MO-345857, MO-345858]!).

= *Mimusops camagueyensis* Urb., Repert. Spec. Nov. Regni Veg. 24: 8. 1927. Type: Cuba. Camagüey, nr. Pastelillo, (fl), *E. Ekman 19071* (lectotype, designated here: S [barcode S13-8677]!; isolectotypes: BM [barcode BM000952453]!, F [barcode V0072101F]!, G [barcode G00439222]!, NY [barcode 00099938]!, S [barcode S-R-7872]!, US [barcode 00113368]!). **(2)**

***Micropholis crassipedicellata* (Mart. & Eichler) Pierre, Not. bot. 40. 1891.**

≡ *Pouteria crassipedicellata* (Mart. & Eichler) Baehni, Candollea. 9: 215. 1942. ≡ *Sideroxylon crassipedicellatum* Mart. & Eichler ex Miq. in Martius, Fl. Bras. 7: 57. 1863. Type: Brazil. Rio de Janeiro: Nr. Canta Gallo, (yfr), *T. Peckolt 356 [336*] (lectotype [first-step designated by Pennington [1990: 201], as “holotype”], second-step designated here: BR [barcode 005416455]!; isolectotypes: BR [barcode 005416783]!, P [barcode P00648170]!). **(2)**

**Note.** Vouchers at BR herbarium have Peckolt #356 as collector number, but one sample, found at P herbarium (P00648170), brings a different number (Peckolt #336) on the type of *Sideroxylon crassipedicellatum*. In addition, both collections at BR and P were collected in the same place and there is no indication of any collector number in the protologue. Main collections of Peckolt are housed at BR and Pierre probably mistakenly transcribed the annotation on P specimen as #336.

***Micropholis egensis* (A.DC.) Pierre in Urban, Symb. Antill. 5 (1): 127. 1904.**

≡ *Sideroxylon egense* A.DC., Prodr. 8: 182. 1844. Type: Brazil. Amazonas: Ega, (fl.), *E. Poeppig 2516* (holotype: G-DC [barcode G00139887]!; isotypes: G [barcodes G00439282, G00439283, G00439284]!, GOET [barcode GOET010927]!, NY [barcodes 00296955, 00842175]!, P [barcodes P00648172, P00648173, P00648174]!, US [barcode 00113310]!, W [barcodes 0067184, 0067185]!). B†, F neg. 4227.

= *Micropholis martiana* Pierre in Urban, Symb. Antill. 5 (1): 126. 1904. Type: Brasil. Amazonas, R. Japura, *C. Martius s.n.* (lectotype, designated here: U [barcode U 0006600 – fragm.]!). **(1)**

= *Sideroxylon ulei* Krause, Verh. Bot. Vereins Prov. Brandenburg 50: 95. 1909. Type: Brazil. Amazonas: R. Jurua, Marary, (fl.), *E. Ule 5162a* (lectotype, designated here: K [barcode K000641477]!; isolectotypes: CORD [barcode CORD00003654]!, G [barcodes G00439281, G00439284]!, GOET [barcode GOET010928]!, IAN [n.v.], RB [barcodes RB00544087, RB00560370]!, W [barcodes 1905-0003203, 1998-0005151]!). B†, F neg. 4229. **(3)**

**Micropholis guyanensis (A.DC.) Pierre subsp. guyanensis , Not. bot. 2: 40. 1891.**

≡ *Sideroxylon guyanense* A.DC., Prodr. 8: 182. 1844. Type: French Guiana. [Cayenne], (fl), *C. Martin s.n.* (holotype: P [barcode P00649234]!; isotypes: G [barcode G00139910]!, P [barcodes P00649235, P00649236]!, US [barcode 00323783]!).

= *Sideroxylon cyrtobotryum* Mart. ex Miq. in Mart., Fl. Bras. 7: 57. 1863. Type: Brazil. Amazonas: nr. mouth of Rio Negro, (fl), *R. Spruce 1530* (lectotype, designated here: K [barcode K000641472]!; isolectotypes: BM [barcodes BM000952609, BM000952610]!, BR [barcode 005416172]!, E [barcode E00205609]!, F [barcodes V0072233F, V0072234F]!, G [barcodes G00439268, G00439269]!, GH [barcode GH00075912]!, GOET [barcode GOET010929]!, K [barcodes K000641471, K000641472]!, NY [barcode 00296953]!, P [barcodes P00649225, P00649226]!, RB [barcodes RB00544083, RB00560365]!, W [barcode 1889-0118439]!). **(2)**

= *Chrysophyllum melinonii* Engl., Bot. Jahrb. Syst. 12: 521. 1890. Type: French Guiana. (fl), *M. Melinon s.n.* (lectotype, designated here: P [barcode P00649231]!; isolectotypes: F [barcode V0071938F,]!, LE [barcode LE00016012]!, MPU [barcodes MPU019047, MPU019048]!, P [barcodes P00649229, P00649230, P00649232]!). B†, F neg. 4250. **(2)**

***Micropholis humboldtiana* (Roem. & Schult.) T.D.Penn., Fl. Neotrop. Monogr. 52: 212–214. 1990.**

≡ *Chrysophyllum humboldtianum* Roem. & Schult., Syst. veg. 4: 813. 1819. Type: America merid., *F. Humboldt & A. Bonpland 1116* (lectotype, designated here: B [barcode BW04593010]!). **(2)**

= *Sideroxylon spruceanum* Mart. & Miq. in Martius, Fl. Bras. 7: 53. 1863. Type: Brazil. Amazonas: R. Negro below Barcellos, (fl.), *R. Spruce 1917* (lectotype, designated here: P [barcode 00649242]!; isolectotypes: BM [barcode BM00603423]!, BR [barcode 005416196]!, E [barcode E00208056]!, F [barcode V0072250F]!, G [barcodes G00439264, G00439265]!, GH [barcode GH00075917]!, K [barcodes K00641495, K00641496]!, NY [barcode 00296963]!, P [barcode P00649243]!, RB [barcodes RB00560371, RB00544086]!). **(2)**

**Note.** The new combination of *Chrysophyllum humboldtianum* as *Micropholis humboldtiana* was performed by Pennington (1990) citing the specimen “Humboldt & Bonpland s.n. (holotype, B-W (herb. n° 4593) n.v.)” with no other additional information. Nevertheless, that specimen mentioned by the author from Willdenow’s collection at B herbarium is actually a folder under #4593 which is composed of three vouchers with collector numbers #1116 (BW04593010), #1117 (BW04593030) and another with no number annotation (BW04593020). After analysing these specimens, we concluded that #1116 might be the corresponding material of *C. humboldtianum* and also that #1117 and the unnumbered specimen are uncertain. Based on that, we selected the voucher Humboldt & Bonpland #1116 (BW04593010) as lectotype.

***Micropholis macrophylla* (Krause) T.D.Penn., Fl. Neotrop. Monogr. 52: 225. 1990.**

≡ *Lucuma macrophylla* Krause, Verh. Bot. Vereins Prov. Brandenburg 50: 94. 1909. Type: Peru. Loreto: Cerro de Escalero, (fl.), *E. Ule 6793* (lectotype, designated here: K [barcode K000641504]!; isolectotypes: CORD [barcode CORD003652]!, F [barcode V0042269F,]!, G [barcode G00237369]!, HBG [barcode HBG510672]!, L [barcode L 0006427]!), MG [n.v.]. B†, F neg. 4191. **(3)**

***Micropholis polita* (Griseb.) Pierre, Not. bot. 41. 1891.**

≡ *Sapota polita* Griseb., Pl. Wright. 2: 517. 1862. Type: Cuba. Orienta, near Monte Verde, (fl.), *C. Wright 1323* (lectotype, designated here: GH [barcode GH00075860]!; isolectotypes: BR [barcode 0000005416851]!, G [barcode G00439261]!, GOET [barcode GOET010932]!, K [barcode K000641468]!, MO [barcode MO-345852]!, P [barcode P00649250]!, YU [barcode YU001710]!). **(2)**

***Micropholis retusa* (Spruce ex Miq.) Eyma, Recueil Trav. Bot. Néerl. 33: 198. 1936.**

≡ *Lucuma retusa* Spruce ex Miq. in Martius, Fl. Bras. 7: 70. 1863. Type: Brazil. Amazonas: Rio Uaupes, near Panure, (fl.), *R. Spruce 2735* (lectotype, designated here: K [barcode K000641475]!; isolectotypes: BM [barcode BM000952608]!, BR [barcode 05416509]!, E [barcode E00208025]!, F [barcodes V781839F, V875622F]!, G [barcodes G00237367, G00237368]!, GH [barcode GH00075649]!, GOET [barcode GOET010933]!, K [barcode K000641476]!, MO [barcode MO-345915]!, NY [barcode 00273519]!, P [barcodes P00649253, P00649254]!, RB [barcodes RB00544028, RB00560355]!). **(2)**

***Micropholis rugosa* (Sw.) Pierre, Not. bot. 41. 1891.**

≡ *Chrysophyllum rugosum* Sw., Prodr. 49. 1788. Type: Jamaica. (yfl.), *O. Swartz s.n.* (holotype: S [barcode S-R-1110]!; isotypes: BM [barcode BM000952614]!, LD [barcode 1260585]!, LINN [barcode HS381-6]!, M [barcode M0174588]!, SBT [barcode SBT12784]!).

= *Chrysophyllum pomiforme* Bertero ex Spreng., Syst. Veg. 1: 667. 1824. Type: Jamaica. *C. Bertero s.n.* (lectotype, designated here: MO [barcode MO-345851]!; isolectotypes: G-DC [barcode G00139917]!, P [barcode P00649256]!). **(2)**

***Micropholis trunciflora* Ducke, Bol. Tecn. Inst. Agron. 19: 19. 1950.**

*Micropholis trunciflora* Ducke, Bol. Tecn. Inst. Agron. 19: 19. 1950. Type: Brazil. Amazonas: Manaus, Estrada do Aleixo, (fl.), *A. Ducke 2216* (holotype: RB [barcode 00544036]!; isotypes: IAN [n.v. – probably the sample was transferred to RB], MG [n.v.], NY [barcode 01200481]!).

= *Pouteria klugii* Baehni, Candollea 14: 76. 1952. Type: Peru. Loreto: Nr. Iquitos, Mishuyacu, (fl), *G. Klug 130* (lectotype, designated here: NY [barcode 00273643]!; isolectotypes: F [barcode V0042279F]!, G [barcode G00439256]!, US [barcode 00067581]!). **(2)**

***Micropholis venulosa* (Mart. & Eichler) Pierre, Not. bot. 40. 1891.**

≡ *Sideroxylon venulosum* Mart. & Eichler, Fl. Bras. 7: 52. 1863. Type: Venezuelan-Colombian frontier. Rio Guainia, near mouth of Rio Casiquiare, (fl., fr.), *R. Spruce 3506* (lectotype, designated here: K [barcode K000641480]!; isolectotypes: BM [barcode BM000952612]!, BR [barcodes 005416202, 005416837]!, C [n.v.], F [barcodes V0072252F – fragm., V0072253F – fragm.]!, G [barcode G00439249]!, M [n.v.], MO [barcode MO-345897]!, MPU [barcode MPU013055]!, NY [barcode 00296964]!, OXF [n.v.], P [barcodes P00649272, P00649273]!, RB [barcodes RB00560369, RB00544088]!, W [barcode 1889-0118452]!). B†, F neg. 4198. **(3)**

**Note.** Type collection is labelled with Spruce #3506; however, most samples also bring #1476. The latter is solely herein included due to its relation on the vouchers’ labels and there is no reference to it on the protologue whatsoever.

***Pouteria campechiana* (Kunth in Humb., Bonpl. & Kunth) Baehni, Candollea 9: 398. 1942.**

≡ *Lucuma campechiana* Kunth in Humb., Bonpl. & Kunth, Nov. Gen. Sp. 3: 240. 1819. Type: Mexico. Nr. Campeche, (fl), *F. Humboldt & A. Bonpland s.n.* (holotype: P [barcode P00670927]!; isotype: MO [barcode MO-1185613]!). B†, F neg. 4186.

= *Lucuma palmeri* Fernald, Proc. Amer. Acad. Arts 33: 87. 1897. Type: Mexico. Guerrero: Acapulco, Oct 1894 – Mar 1895, (fl), *E. Palmer 386* (lectotype, designated here: US [barcode 00113263]!; isolectotypes: F [barcodes V0072005F, V0072006F, V0072007F – mounted on two herbarium sheets]!, K [barcode K000641066]!, MO [mounted on two herbarium sheets, barcodes MO-157709, MO-157710]!, NY [barcode 00273490]!, U [barcode U 0006634]!, UC [barcode UC125678]!). **(1)**

***Pouteria cayennensis* (A.DC.) Eyma, Recueil Trav. Bot. Neerl. 33: 174. 1936.**

≡ *Richardella cayennensis* (A.DC.) Aubrév., Adansonia 11: 300. 1971. ≡ *Chrysophyllum cayennense* A.DC. in A. P. de Candolle, Prodr. 8: 160. 1844. Type: French Guiana. [Cayenne], (f1), *J. Martin s.n.* (lectotype [first-step designated by Pennington [1990: 366], as “holotype”], second-step designated here: P [barcode P00640549]!; isolectotypes: G-DC [barcode G00139668]!, P [barcodes P00640548, P00640550]!, US [barcode 00037030]!). **(2)**

= *Chrysophyllum sessiliflorum* Poir., Encycl. suppl. 2: 16. 1811. Type: French Guiana. [Cayenne], (f1), *J. Martin s.n.* (holotype: P [barcode P00640551]!).

= *Lucuma pulverulenta* Mart. & Eichler in Martius, Fl. Bras. 7: 70. 1863. Type: French Guiana. [Cayenne], (f1), *J. Martin s.n.* (lectotype designated here: P [barcode P00640545]!; isolectotypes: BM [barcode BM00952534]!, F [barcodes V0072057F, V0072058F – fragm.]!, NY [barcode 00273518]!, P [barcode P00640544]!). B†, F neg. 4196. **(2)**

**Note.** When considered conspecific, under the genus *Pouteria*, *Chrysophyllum sessiliflorum* Poir. would have priority over *C. cayennense* A.DC. However, as the combination *Pouteria sessiliflora* (Sw.) Poir. was already occupied, the next earliest epithet is to be used, as done by Eyma (1936), who published the new combination *Pouteria cayennensis* (A.DC.) Eyma. In the protologue of *Chrysophyllum cayennense*, De Candolle (1844) provided the information “In Guyana prope Cayennam” and “v. s. comm. a Mus. par.”, but he did not make any reference to the collector’s name or number. When transferring *C. cayennense* to *Pouteria cayennensis*, Eyma (1936) assumed Martin’s collection s.n. at P herbarium as type. However, Baehni (1942) cited the type material was housed in the B herbarium as it follows “Guyane française; Cayenne (Martin s.n. !! = type in hb. B.)”. Nevertheless, after consulting both herbaria (B and P), we found three specimens at P and none at B. A little while after De Candolle’s publication, Martius & Eichler in Miquel (1863) described *Lucuma pulverulenta*, based on Martin’s material, probably from the herbarium B. Pennington (1990) cited “holotype, P” for *Chrysophyllum cayennense*, thereby inadvertently lectotypifying the name with a sheet at P. However, there are three different vouchers and we choose P (P00640549) as lectotype.

***Pouteria cinnamomea* Baehni, Candollea. 9: 252. 1942.**

≡ *Labatia discolor* Diels in Engl. Bot. Jahrb. Syst. 37: 601. 1906. Type: Peru. Cuzco: Prov. Convención, Idma, nr. Santa Anna, Jul 1905 (fl), *A. Weberbauer 5034* (lectotype, designated here: G [barcode G00439397]!; isolectotypes: F [barcodes V0042264F – fragm., V0042265F – fragm., V0042266F – fragm.]!). B†, F neg. 4232. **(3)**

**Pouteria dictyoneura subsp. fuertesii (Urb.) Cronquist, Lloydia 9(4): 267. 1946.**

≡ *Paralabatia fuertesii* Urb., Symb. Antill. 7: 323. 1912. Type: Dominican Republic. Nr. Barahona, May 1910 (fl), *M. Fuertes 19* (lectotype, designated here: S [barcode S05-2296]!; isolectotypes: BM [n.v.], BR [barcode 005415403]!, F [barcode V0072119F]!, GH [barcode GH00075779]!, GOET [barcodes GOET010939, GOET010940]!, HBG [barcode HBG510713]!, K [barcode K000641075]!, L [barcodes L 0006323, L 0006324]!, M [barcode M0174271]!, MO [barcode MO-2049469]!, SI [barcode SI003223]!, US [barcode 00113280]!). **(2)**

***Pouteria elegans* (A.DC.) Baehni, Candollea 9: 197. 1942.**

≡ *Sideroxylon elegans* A.DC. in A. P. de Candolle, Prodr. 8: 183. 1844. Type: Brazil. Amazonas: Ega, Sep 1831 (fl), *E. Poeppig 2492* (holotype: G-DC [barcode G00139949]!; isotypes: F [barcodes V0072241F, V0072242F – fragm.]!, GOET [barcode GOET010941]!, HAL [barcode 0139344]!, NY [barcode 00296956]!, OXF [n.v.], P [barcodes P00640583, P00640584]!, US [barcode 00113311]!, W [barcodes 0066769, 1889-0129955]!). B†, F neg. 4218.

= *Pouteria arbuscula* Baehni, Candollea 14: 75. 1952. Type: Brazil. Amazonas: Basin of R. Negro San Gabriel, Sep 1928 (fl), *G. Tate 141* (lectotype, designated here: US [barcode 00113273]!; isolectotypes: G [barcode G00439480]!, NY [barcode 01211558]!). **(3)**

***Pouteria gardneriana* (A.DC.) Radlk., Sitzungsber. Math.-Phys. Cl. Konigl. Bayer. Akad. Wiss. München 12: 333. 1882.**

≡ *Lucuma gardneriana* A.DC. in A. P. de Candolle, Prodr. 8: 168. 1844. Type: Brazil. Piaui: Betw. Angrias & Sao Gonsalvo, Feb 1839 (fl), *G. Gardner 2228* (holotype: G [barcode G00439465]!; isotypes: BM [barcodes BM00952559, BM00952560]!, G-DC [barcode G00439466]!, GH [barcode GH00075638]!, K [barcodes K000641158, K000641159]!, NY [barcode 00273500]!, OXF [n.v.], P [barcodes P00647918, P00647919]!, W [barcode 1889-0092115]!. B†, F neg. 4202.

= *Labatia ciliolata* Engl., Bot. Jahrb. Syst. 12: 515. 1890. Type: Brazil. Santa Catarina: Blumenau, Oct 1886 (fl), *J. Schenck 907* (lectotype [first-step designated by Pennington [1990: 451], as “isotype”], second-step designated here: P [barcode P00647921]!; isolectotype: P [barcode P00647920]!). B†, F neg. 4231. **(3)**

= *Lucuma lanceolata* Raunk., Vidensk. Meddel. Dansk Naturhist. Foren. Kjøbenhavn: 8. 1889. Type: Brazil. ? Rio de Janeiro, *A. Glaziou 14057a* (lectotype, designated here: P [barcode P00647922]!; isolectotypes: BR [barcode 005334988]!, C [barcodes C10018773, C10018774]!, G [barcode G00439463]!, K [barcode K000641156]!, MO [barcode MO-1994445]!, NY [barcode 00375397]!, P [barcodes P00647923, P00647924]!, US [barcode 00323781]!). **(1)**

**Pouteria glomerata subsp. stylosa (Pierre) T.D.Penn., Fl. Neotrop. Monogr. 52: 420–422. 1990.**

≡ *Guapeba stylosa* Pierre, Not. bot. 2: 42. 1891. Type: Panama. Obispo Falls nr. P.R.R. (fl), *S. Hayes 67* (holotype: BR [barcode 0000005415083]!; isotypes: BM [barcode BM000645679]!, E [barcodes E00259468, E00259469]!, G [barcode G00439455]!, K [n.v.], M [barcode M0174351 – fragm.]!, P [barcodes P00647929, P00647930]!, W [n.v.].

= *Pouteria cuprea* Huber, Bull. Soc. Bot. Geneve, sér.2, 6: 198. 1914. Type: Brazil. Pará: R. January, Prainha, May 1903 (fl), *A. Ducke s.n.* (MG n° 3568) (lectotype, designated here: RB [barcode RB00544050]!; isolectotype: BM [barcode BM000952563]!). **(1)**

***Pouteria gomphiifolia* (Mart.) Radlk., Sitzungsber. Math.-Phys. Cl. Konigl. Bayer. Akad. Wiss. Miinchen 12: 33. 1882.**

≡ *Lucuma gomphiifolia* [“*gomphiaefolia*”] Mart. ex Miq. in Martius, Fl. Bras. 7: 78, tab. 37, fig. 1. 1863. Type: Brazil. Amazonas: Mouth of R. Negro, N bank of Amazon, Aug 1851 (fl), *R. Spruce 1640* (lectotype, designated here: K [barcode K000641421]!; isolectotypes: BM [barcodes BM000952557, BM000952558]!, BR [barcode 0005415410]!, E [barcode E00259465]!, F [barcodes V0072033F – fragm., V0072034F – fragm.]!, G [barcode G00439453]!, GH [barcode GH00075608]!, GOET [barcode GOET010945]!, K [barcode K000641419]!, NY [barcodes 01211847, 00273502]!, OXF [n.v.], P [barcodes P00647931, P00647932]!, RB [barcode RB00378876]!, W [barcodes 1889-0092125, 1889-0118421]!). **(2)**

**Note.**Pennington (1990) cited Spruce #1670 [1640] as type material of *Lucuma gomphiifolia* as a reflex of the available information on the herbaria database. That is a mistake once Martius ex Miquel (1863) clearly informed Spruce #1640 as type collection of *Lucuma gomphiifolia*. In addition, Spruce #1670 corresponds to the lectotype of *Passiflora costata* Mast. (Passifloraceae) (Masters, 1872). Furthermore, other samples under Spruce #3117 (1670), or even only Spruce #3117, refer to the type collection of Lucuma gomphiifolia var. blepharanta Mart. (= *Pouteria gomphiifolia* (Mart.) Radlk.).

***Pouteria longifolia* (Mart. & Eichler) T.D.Penn.**

≡ *Chrysophyllum longifolium* Mart. & Eichler in Martius, Fl. Bras. 7: 97. 1863. Type: Peru. San Martín: Tarapoto, Oct 1865 (yfr), *R. Spruce 4234* (lectotype, designated here: K [barcode K000641453]!; isolectotypes: BM [barcode BM000952527]!, BR [barcode 005415120]!, K [barcode K000641452]!, P [barcode P00647954]!). **(2)**

***Pouteria lucens* (Mart. & Miq.) Radlk., Sitzungsber. Math.-Phys. Cl. Königl. Bayer. Akad. Wiss. München 12: 333. 1882.**

≡ *Lucuma lucens* Mart. & Miq. in Mart., Fl. Bras. 7: 78. 1863. Type: Brazil. Amazonas: R. Vaupés, nr. Panure, *R. Spruce 2504* (lectotype, designated here: K [barcode K000641422]!; isolectotypes: A [n.v.], BM [barcode BM000952555]!, BR [barcode 005415168]!, E [barcode E00259464]!, F [barcode V0072043F – fragm.]!, G [barcodes G00439542, G00439543, G00439544, G00439545]!, GH [barcode GH00075643]!, GOET [barcode GOET010946]!, K [barcode K000641422]!, OXF [n.v.], MO [barcode MO-391907]!, NY [barcode 00273509]!, P [barcodes P00647955, P00647956]!, RB [barcodes RB00560332, RB00544020]!, W [barcodes 1889-0118424, 1889-0092126]!). **(1)**

***Pouteria lucuma* (Ruiz & Pav.) Kuntze, Revis. gen. pl. 3(2): 195. 1898.**

≡ *Achras lucuma* Ruiz & Pavón, Fl. peruv. 3: 17, t. 239. 1802. Type: Peru. Without exact locality, (fl), *J. Pavón s.n.* (lectotype, designated here: G [barcode G00439536]!; isolectotypes: G [barcodes G0043953, G00439537, G00439538, G00439540]!, MA [barcode MA-CARPO-100101]!). **(1)**

***Pouteria mattogrossensis* (Pilg.) Baehni, Candollea 9: 238. 1942.**

≡ *Labatia mattogrossensis* Pilg., Bot. Jahrb. Syst. 30: 181. 1902. Type: Brazil. Mato Grosso: Upper Kulisehu, Jul 1899 (fl), *R. Pilger 736* (lectotype, designated here: B [barcode B100248112]!; isolectotypes: F [barcode V0071982F – fragm.]!, NY [n.v.], US [n.v.]). B†, F neg. 4233. **(3)**

***Pouteria moaensis* Alain, Mem. Soc. Cub. Hist. Nat. “Felipe Poey” 22: 113. 1955.**

*Pouteria moaensis* Alain, Mem. Soc. Cub. Hist. Nat. “Felipe Poey” 22: 113. 1955. Type: Cuba. Oriente, Moa, E of airstrip, Jun 1945 (fl), *B. Clemente 4389* (lectotype, designated here: NY [barcode 00099949]!; isolectotypes: A [n.v.], GH [barcode GH00075815]!, US [barcode 00037035]!. **(1)**

***Pouteria obscura* (Huber) Baehni, Candollea 9: 361. 1942.**

≡ *Lucuma obscura* Huber, Bull. Soc. Bot. Genève, sér. 2, 6: 196, fig 9. 1914. Type: Brazil. Pará: Lower Trombetas, NE of Cuminá-mirim, Dec 1906 (fl), *A. Ducke s.n.* (MG n° 7974) (lectotype, designated here: RB [barcode RB00544021]!; isolectotype: BM [barcode BM000952547]!). **(1)**

***Pouteria psammophila* (Mart.) Radlk., Sitzungsber. Math.-Phys. C1. Konigl. Bayer. Akad. Wiss. Minchen 12: 333. 1882.**

≡ *Labatia psammophila* Mart., Flora 21 (2), Beibl. 2 (4): 93. 1838 (reprinted as Herb. Fl. Bras. 173). Type: Brazil. Rio de Janeiro: Cabo Frio, (fl), *M. Wied-Neuwied s.n.* (holotype: BR [barcode 0006590642]!; isotypes: MEL [barcodes MEL 2353766A, MEL 2353767A]!, U [barcode U 0006685]!).

= *Pouteria crassinervia* Engl., Bot. Jahrb. Syst. 12: 514. 1890. Type: Brazil. Rio de Janeiro: Cabo Frio, *J. Schenck 3920* (lectotype, designated here: P [barcode P00648028]!; isolectotype: F [barcode V0072157F – fragm.]!). B†, F neg. 4200. **(3)**

***Pouteria ramiflora* (Mart.) Radlk., Sitzungsber. Math.-Phys. Cl. Konigl. Bayer. Akad. Wiss. Miinchen 12: 333. 1882.**

≡ *Labatia ramiflora* Mart., Flora 21 (2), Beibl. 2 (4): 93. 1838. (reprinted as Herb. Fl. Bras. 173). Type: Brazil. Minas Gerais: “inter vicum Contendas et praedium Tamanduain, deserto Prov. Minarum,” Aug 1818 (fl), *C. Martius s.n.* (holotype: M [barcode M0174362]!; isotypes: M [barcodes M0174363, M0174366, M0174367]!).

= *Lucuma lateriflora* Benth. ex Miq. in Martius, Fl. Bras.7: 83. 1863. Type: Brazil. Para: Nr. Santarem, Jul 1850 (yfl), *R. Spruce 728* (lectotype, designated here: E [barcode E00259460]!; isolectotypes: E [barcode E00259461]!, GH [barcode GH00075642]!, GOET [barcode GOET010951]!, M [barcode M0174359]!, NY [barcode 00273508]!, S [barcode S05-4709]!), U [barcode U 0006690]!. **(2)**

= *Lucuma parviflora* Benth. ex Miq. in Martius, Fl. Bras. 7: 81, tab. 34. 1863. Type: Brazil. Pará: Nr. Santarém, Jul 1850 (fl), *R. Spruce 729* (lectotype, designated here: E [barcode E00259462]!; isolectotypes: E [barcode E00259463]!, K [barcode K000641105]!, M [barcode M0174358]!, NY [barcode 00273514]!, P [barcode P00648035]!, U [barcode U 0006691]!). **(2)**

= Pouteria ramiflora var. grandifolia Kuntze, Revis. gen. pl. 3(2): 195. 1898. Type: Brazil. Mato Grosso, Jul 1892 (fl), *C. Kuntze s.n.* (lectotype, designated here: NY [barcode 00860139]!; isolectotypes: F [barcode V0072186F, V0072187F – fragm.]!, G [barcode G00439502]!, MO [n.v.]). **(1)**

= Pouteria ramiflora var. oblongifolia Kuntze, Revis. gen. pl. 3(2): 195. 1898. Type: Bolivia. Velasco, Jul 1892 (fl), *C. Kuntze s.n. [55*] (lectotype, designated by Pennington [1990: 279], as “isotype”: F [barcode V0072188F]!).

**Note 1.**Pennington (1990) and available information on the herbaria database cited Spruce #926 (728) and Spruce #926 (729) as type materials of *Lucuma lateriflora* and *Lucuma parviflora* Bentham ex Miq., respectively. However, Bentham in Miquel (1863) clearly cited in the protologue only Spruce #728 and #729 for them. In addition, specimens Spruce #926 correspond to *Sematophyllum inundatum* Mitt. (= *Trichosteleum inundatum* (Mitt.) A. Jaeger) (Sematophyllaceae – Bryophyta).

**Note 2.** There is no type citation in the protologue of Pouteria ramiflora var. grandifolia and, according to Zanoni (1980), Kuntze’s main set of plant collection is currently housed at NY. Thus, we selected the voucher NY [barcode 00860139], which is well-preserved and exhibits flowers and floral buds, as lectotype.

**Pouteria rigida (Mart. & Eichler) Radlk. subsp. rigida , Sitzungsber. Math.-Phys. Cl. Königl. Bayer. Akad. Wiss. München 12: 333. 1882.**

≡ *Lucuma rigida* Mart. & Eichler in Martius, Fl. Bras. 7: 73. 1863. Type: Guyana. Nr. Roraima, 1842, *Rich. Schomburgk 976* (lectotype, designated by Pennington [1990: 357], as “isotype”: K [n.v.]; isolectotype: F [barcode V0360316F – fragm.]!).

**Note.** There are three different type collections spread in the herbaria under Schomburgk #976. They correspond to *Licania laxiflora* Fritsch (Chrysobalanaceae) (BM (BM000602323, BM000560075), K (n.v.), P (P00746021, P00746022), W (n.v.)) from Robert Schomburgk’s collection [ser. 2] 976, to *Oreodaphne gracilis* Meisn. (= *Ocotea gracilis* (Meisn.) Mez) (Lauraceae) (B (B100185322), BM (BM000993951)) from Robert Schomburgk’s collection [ser. 1] 976 and, lastly, to *Lucuma rigida* Mart. & Eichler (K (n.v.), F (V0360316F)) from Richard Schomburgk’s collection 976 (van Dam 2002). They were cited by their respective protologues and by having information on van Dam (2002). They can be easily distinguished and there is no misunderstanding whatsoever. Concerning *Lucuma rigida*, once Pennington (1990) indicated the sample at K as isotype, he inadvertently lectotypified the name. However, we did not track the voucher at K herbarium and its confirmation is needed.

***Pouteria rostrata* (Huber) Baehni, Candollea 9: 270. 1942.**

≡ *Lucuma rostrata* Huber, Bull. Soc. Bot. Genève, Ser. 2, 6: 195. 1914–1915. Type: Brazil. Para: Lower Trombetas, R Cumina-mirim, Dec 1906 (fl), *A. Ducke s.n.* (MG n° 7968) (lectotype, designated by Pennington [1990: 310], as “isotype”: BM [barcode BM000624871]!; isolectotypes: F [barcode V0072061F – fragm.]!, G [barcode G00439569]!, MG [n.v.], RB [barcode RB00544029]!, US [barcode 00113267]!).

**Note.** The protologue of *Lucuma rostrata* brings specimens under A. Ducke #7968 as type collection. Baehni (1942), based on those specimens, published the new combination, *Pouteria rostrata*, but he mentioned Huber #7968 in the publication as type material. This misunderstanding concerns just the names of collectors because, in both protologue and specimens, the collector is Ducke.

***Pouteria salicifolia* (Spreng.) Radlk., Sitzungsber. Math.-Phys. Cl. Königl. Bayer. Akad. Wiss. München 12: 333. 1882.**

≡ *Roussea salicifolia* Spreng., Syst. veg. 1: 419. 1824. Type: Uruguay. Montevideo [Brazil. without precise locality, without date], [1821–1829], *F. Sellow s.n.* (lectotype, designated here: P [barcode P00648052]!; isolectotypes: L [barcodes L 0820131, L 0820132]!, K [barcodes K000641414, K000641415, K000717694]!, P [barcodes P00648051, P00648053]!). **(2)**

= *Lucuma sellowii* A.DC. in A.P. de Candolle, Prodr. 8: 167. 1844. Type: Brazil [Southern]. Rio Negro [propé Bagé, in Brasil austr.], [1829], *F. Sellow 1727* (lectotype, designated here: BR [barcode 000005580651]!; isolectotypes: F [barcode V0072062F]!, G [n.v.], P [barcode P00648050]!, US [barcode 00067597]!, W [n.v.]). **(3)**

**Note.**Sprengel (1824) cited “*R. foliis lineari-lanceolatis elongatis integerrimis glabris. Monte Video. Sello.*”. However, no specimen was found at any of the herbaria consulted by us. In addition, Pennington (1990) considered this type collection as dubious material “*Roussea salicifolia Spreng., Syst. veg. 1: 419. 1825. Type: Uruguay, Montevideo, Sello s.n. (? isotype P).*”. Under these circumstances, we interpret that Pennington referred to it as untraced voucher. As the type collection with the label cited by Sprengel (1824) was not found, which probably contained locality information, we inferred that those samples kept at L (two sheets), K (three sheets) and P (three sheets) herbaria correspond to the original material. Thus, we selected the specimen at P (P00648052) as lectotype.

***Pouteria surumuensis* Baehni, Candollea 9: 362. 1942.**

≡ *Lucuma sericea* K.Krause, Notizbl. Königl. Bot. Gart. Berlin 6: 169. 1914. nom. illegit. Not *Lucuma sericea* Benth. & Hook. (1876, Australia). Type: Brazil. Roraima: Rio Branco, Serra do Mel, Surumu, Aug 1909, (fl), *E. Ule 8258* (lectotype, designated here: RB [barcode RB00544030]!; isolectotypes: F [barcode V0072198F]!, G [barcode G00439558]!, K [barcode K000641140]!, L [barcode L 0820130]!, U [barcode U 0006700]!). B†, F neg. 4197. **(3)**

**Pradosia schomburgkiana (A.DC.) Cronquist subsp. schomburgkiana , Bull. Torrey Bot. Club 73: 311. 1946.**

≡ *Chrysophyllum schomburgkianum* A.DC. in A. P. de Candolle, Prodr. 8: 157. 1844. Type: Guiana. (fl.), *Rob. Schomburgk [ser. 1] 505* (holotype: G-DC [barcode G00139569]!; isotypes: BM [barcode BM000952571]!, BR [barcode 005415137]!, E [barcode E00259450]!, F [barcodes V0071946F, V0071947F]!, G [barcodes G00439595, G00439596]!, K [barcodes K000640445, K000640446]!, L [barcode L 0820129]!, NY [barcode 00902225]!, OXF [n.v.], P [barcode P00649452]!, U [barcode U 0006718]!, US [barcode 00113129]!, W [n.v.]).

= *Chrysophyllum inophyllum* Mart. ex Miq. in Martius, Fl. Bras. 7: 105. 1863. Type: Brazil. Amazonas: Barra, (fl.), *R. Spruce 1393* (lectotype, designated here: M [barcode M0174519]!; isolectotypes: BM [barcode BM00952568]!, G [barcodes G00439592, G00439593]!, GH [barcodes GH00075576, GH00075577]!, GOET [barcodes GOET010957, GOET010958]!, K [barcodes K000640442, K000640443, K000640444]!, NY [barcode 00273429]!, P [barcodes P00649453, P00649454, P00649455, P00649456]!, RB [barcodes RB00544000, RB00642331, RB00642342]!). **(1)**

***Sideroxylon americanum* (Mill.) T.D.Penn., Fl. Neotrop. Monogr. 52: 118. 1990.**

≡ *Maurocenia americana* Mill., Gard. Dict. (ed. 8) 4: 1768. Type: Jamaica. Palisadoes, *W. Houston s.n.* (holotype: BM [barcode BM000952515]!).

= *Bumelia oblongata* Urb., Symb. Antill. 6: 31. 1909. Type: Jamaica. St. Ann., between Salem & Llandovery, (fr), *W. Harris 10380* (lectotype, designated here: NY [barcode 00099912]!; isolectotypes: BM [barcode BM000952516]!, F [n.v.], K [barcode K000641550]!, US [barcode 01113661]!). **(2)**

= *Bumelia excisa* Urb., Repert. Spec. Nov. Regni Veg. 13: 471. 1915. Type: Jamaica. Pedro Bluff, (fr), *W. Harris 9729* (lectotype, designated here: NY [barcode 00099924]!; isolectotypes: F [n.v.], K [barcode K000641549]!, US [barcode 01113660]!). **(2)**

= *Bumelia navassana* Urb. & Ekman, Ark. Bot. 21A(17): 71. 1929. Type: Haiti. [Between Haiti and Jamaica]. Navassana Island, (fl, fr), *E. Ekman H10811* (lectotype, designated here: S [barcode S09-43780]!; isolectotypes: A [barcode A00075540]!, C [barcode C10018764]!, F [barcode V0071900F]!, G [barcode G00439653]!, GH [barcode GH00075539]!, K [barcode K000641548]!, LL [barcode LL00372382]!, MO [barcodes MO-2000302, MO-859995]!, NY [barcode 00099931]!, P [barcode P00689866]!, S [barcode S-R-9010]!, U [barcode U 0006727]!, US [barcodes 00113097, 00782598]!). **(2)**

***Sideroxylon angustum* T.D.Penn., Fl. Neotrop. Monogr. 52: 147. 1990.**

≡ *Bumelia revoluta* Urb., Symb. Antill. 9: 417. 1925. Type: Cuba. Sierra de Nipe, nr. Woodfred, (fl), *E. Ekman 15261* (lectotype, designated by Pennington [1990: 147], as “isotype”]: NY [barcode 00099917]!; isolectotypes: S [barcode S-R-7864]!, UPS [barcode V-557127]!).

***Sideroxylon anomalum* (Urb.) T.D.Penn., Fl. Neotrop. Monogr. 52: 123. 1990.**

≡ *Dipholis anomala* Urb., Symb. Antill. 7: 325. 1912.

≡ *Bumelia integra* Cronquist, J. Arnold Arbor. 26: 469. 1945. nom. superf. Type: Dominican Republic. Barahona: El Hoyo, (fl), *M. Fuertes 1039* (lectotype, designated here: BM [barcode BM000952514]!; isolectotypes: A [barcode A0075533]!, E [barcode E00205607]!, F [barcodes V0071958F, V0071959F]!, GH [n.v.], HBG [barcode HBG510665]!, K [barcode K000641555]!, L [barcode L 0544796]!, MIN [barcode MIN1000838]!, NY [barcode 00099903]!, P [barcode P00644476]!, S [barcode S05-2116]!, U [barcode U 0006728]!, US [barcodes 00782607, 00113353]!, W [n.v.]). **(2)**

***Sideroxylon dominicanum* (Whetstone & T.A.Atk.) T.D.Penn., Fl. Neotrop. Monogr. 52: 148–149. 1990.**

≡ *Bumelia dominicana* Whetstone & T.A.Atk., Sida 11(4): 396. 1986.

≡ *Bumelia ferruginea* (Ekman & O.C.Schmidt) Stearn, J. Arnold Arbor. 49: 287. 1968. nom. illeg.

≡ *Dipholis ferruginea* Ekman & O.C.Schmidt, Repert. Spec. Nov. Regni Veg. 32: 94. 1933. Type: Dominican Republic. Province Samana: Los Haitises, Boca del Infierno, (fl), *E. Ekman H15406* (lectotype, designated here: S [barcode S05-2124]!; isolectotypes: A [barcode A0075597]!, F [barcode V0071960F]!, G [barcode G00439639]!, GH [barcode GH00075596]!, K [barcode K000641570]!, NY [barcode 00099905]!, US [barcode 00113355]!). **(2)**

***Sideroxylon horridum* (Griseb.) T.D.Penn., Fl. Neotrop. Monogr. 52: 127. 1990.**

≡ *Bumelia horrida* Griseb., Cat. pl. Cub. 165. 1866. Type: Cuba. (fl), *C. Wright 2922* (holotype: GOET [barcode GOET010964]!; isotypes: BM [barcode BM00952512]!, G [barcodes G00439633, G00439634]!, GH [barcode GH00075532]!, K [barcode K000641559]!, MO [n.v.], NY [barcodes 1443596, 1443559]! P [barcodes P00644521, P00644522, P00644523, P00644524, P00644525]!, US [barcode 00113096]!, W [n.v.], YU [barcode YU001714]!).

= *Bumelia glomerata* Griseb., Pl. Wright 2: 518. 1862. Type: Cuba. (fl), *C. Wright 347* (lectotype, designated here: GH [barcode GH00075530]!; isolectotypes: BR [barcode 005334957]!, G [barcodes G00439631, G00439632]!, K [barcode K000641557]!, NY [barcode 00099925]!, P [barcode P00644528]!). **(2)**

***Sideroxylon jubilla* (Ekman ex Urb.) T.D.Penn., Fl. Neotrop. Monogr. 52: 137. 1990.**

≡ *Dipholis jubilla* Ekman ex Urb., Symbol. Antill. 9: 415. 1925.

≡ *Bumelia jubilla* (Ekman ex Urban) Stearn, J. Arnold Arbor. 49: 287. 1968. Type: Cuba. Oriente: Alto de Iberia, Nov 1916 (fl), *E. Ekman 8324* (lectotype, designated here: S [barcode S-R-7869]!; isolectotypes: A [barcode A00075598]!, BM [barcode BM00952506]!, F [barcode V0071961F]!, G [barcode G00439628]!, NY [barcode 00099896]!, UPS [barcode V-557261]!). **(2)**

***Sideroxylon montanum* (Sw.) T.D.Penn., Fl. Neotrop. Monogr. 52: 137. 1990.**

≡ *Bumelia montana* Sw., Prodr. 49. 1788. Type: Jamaica. (fl), *O. Swartz s.n.* (holotype: S [barcode S-R-780]!; isotypes: G [barcode G00439623]!, M [barcode M0174567]!, P [barcode P00644531]!).

= *Dipholis pallens* Pierre & Urb., Symbol. Antill. 5: 136. 1904. Type: Jamaica. Blue Mountains, Iron Face, Chester Vale, (fl), *W. Harris 5340* (lectotype, designated here: P [barcode P00644530]!; isolectotypes: BM [barcode BM000952505]!, NY [barcode 00099899]!). **(2)**

***Sideroxylon obovatum* Lam., Tabl. Encycl. 2: 42. 1974. Type: America merid.?, *collector s.n.* (holotype: P-LA [n.v.]).**

= *Bumelia heterophylla* Urb., Symb. Antill. 7: 326. 1912. Type: Dominican Republic. nr. Constanza, (fl), *H. von Türckheim 3473* (lectotype, designated here: BR [barcode 0000005416462]!). **(3)**

= *Bumelia lineolata* Urb. & Ekman, Ark. Bot. 21A(5): 56. 1927. Type: Haiti. Massif de la Selle, nr. Ansesa-Pitre, (fl), *E. Ekman 6968* (lectotype, designated here: S [barcode S05-2164]!; isolectotypes: K [barcode K000641541]!, NY [barcode 00099928]!, S [barcode S05-2165]!). **(2)**

**Sideroxylon obtusifolium subsp. buxifolium (Roem. & Schult.) T.D.Penn., Fl. Neotrop. Monogr. 52: 116. 1990.**

≡ *Bumelia buxifolia* Roem. & Schult., Syst. veg. 4: 802. 1819. Type: Venezuela. Sucre: Cumana, (fl), *F. Humboldt & A. Bonpland s.n. [561*] (holotype: B-W [barcode BW04603010]!; isotypes: BM [n.v.], P [barcode P00670923]!.

= *Bumelia nicaraguensis* Loes., Bot. Jahrb. Syst. 60: 367. 1926. Type: Nicaragua. Matagalpa: between Esquipulos y San Dionisio, (fl, fr), *E. Rothschuh 463* (lectotype, designated by Pennington [1990: 116], as “isotype”: F [n.v.]). B†, F neg. 4275.

***Sideroxylon peninsulare* (Brandegee) T.D.Penn., Fl. Neotrop. Monogr. 52: 105. 1990.**

≡ *Bumelia peninsularis* Brandegee, Zoë 5: 107. 1900. Type: Mexico. Baja California: Sierra de la Laguna, (fl), *T. Brandegee s.n.* (lectotype, designated here: GH [barcode GH00075506]!; isolectotypes: US [barcodes 02703605, 00344806]!). **(2)**

***Sideroxylon picardae* (Urb.) T.D.Penn., Fl. Neotrop. Monogr. 52: 126. 1990.**

≡ *Bumelia picardae* Urb., Symb. Antill. 5: 148. 1904. Type: Haiti. Plain, (fl), June 1894, *L. Picarda 1242* (lectotype, designated here: P [barcode P00644571]!; isolectotypes: GH [barcode GH00075542]!, NY [barcode 00099913]!). **(2)**

***Sideroxylon repens* (Urb. & Ekman) T.D.Penn., Fl. Neotrop. Monogr. 52: 131. 1990.**

≡ *Dipholis repens* Urb. & Ekman, Ark. Bot. 22A(17): 70. 1929. Type: Dominican Republic. Prov. Barahona: Cordillera de Bahoruco, (fl), *E. Ekman H6790* (lectotype, designated here: S [barcode S05-2136]!; isolectotypes: A [n.v.], G [barcode G00439664]!, GH [barcode GH00075599]!, K [barcode K000641566]!, NY [barcode 00099900]!, S [barcode S05-2139]!, US [barcodes 00113356, 00782524]!). **(2)**

***Sideroxylon rotundifolium* (Sw.) T.D.Penn., Fl. Neotrop. Monogr. 52: 120. 1990.**

≡ *Bumelia rotundifolia* Sw., Prodr. 50. 1788. Type: Jamaica. (fl), *O. Swartz s.n.* (holotype: S [barcode S-R-784]!; isotype: M [barcode M0174381]!).

= *Bumelia purdiaei* Urb., Symb. Antill. 5: 143. 1904. Type: Jamaica. St. Catherine: banks of River Cobre, (fl), *W. Purdie s.n.* (lectotype, designated here: K [barcode K000641551]!; isolectotypes: K [barcode K000641552]!, NY [barcode 00099914]!). **(2)**
